# Change during Psychotherapy through Sand Play Tray in Children That Have Been Sexually Abused

**DOI:** 10.3389/fpsyg.2017.00617

**Published:** 2017-05-04

**Authors:** María D. L. Angeles Tornero, Claudia Capella

**Affiliations:** Department of Psychology, Universidad de ChileSantiago, Chile

**Keywords:** psychotherapy, sexual abuse, children, play, sandplay

## Abstract

This paper presents the results of a qualitative study on the use of sandplay, or sand tray therapy, in the psychotherapeutic process of children who have been sexually abused. A longitudinal study was carried out with seven participants between the ages of 7 and 10 years old. Data was produced during observation of the therapeutic activity over the course of three different phases of treatment, using a rubric created especially for this observation. Three sandplay sessions were recorded: one at the start of therapy, one at the 3-month mark, and the third and final session after 6 months of treatment. Sessions were then transcribed for later analysis. A rubric was developed in order to help researchers identify central themes, behaviors and content of creative play, as well as the therapeutic relationship. Transcribed sessions and observation rubrics were evaluated using qualitative content analysis, and information was categorized according to verbal and behavioral characteristics of the game. Results of the present study reveal common and transversal forms of playful expression among this group of children shown by their engagement with sandplay. During this activity, participants elaborate personal stories that feature violence as a central theme, often involving aggression between two or more individuals. They also express their need for care and protection and work to resolve conflicts using fantasy. The shifting dynamics of sandplay at each stage of therapeutic treatment is an important finding that reveals the progress made during psychotherapy. In the third phase of treatment, sandplay encouraged movement among children, allowing them to act out meaningful scenarios and create structured situations with positive outcomes. Finally, the value of sandplay as an important therapeutic tool is discussed, primarily its role in supporting processes of change and allowing participants to assign new meanings to traumatic experiences. Its application to the field of clinical psychology, particularly when working with victims of sexual abuse, is also explored.

## Introduction

Child sexual assault is a serious issue which has drawn increasing international attention in recent years. Studies show that nearly 150 million girls and 73 million boys under 18 years have experienced sexual abuse (United Nations Children’s Fund [UNICEF], 2006). In Chile, studies show that around 7% of boys and girls between 11 and 17 years old have suffered from sexual abuse at some point in their lives (Ministerio del Interior [Home Office], 2013). These statistics have prompted debates from diverse sectors of society on how to best analyze and strengthen legislation toward eradicating child abuse.

Many different authors from across the world have argued that sexual assault may profoundly impact the life of a child by interfering with their psychological functioning and development (Echeburúa and Corral, 2006; Cutajar et al., 2010). Thus, one of the most widely used models applied to cases of sexual assault and their consequences, proposed by Finkelhor and Browne (1985), posits four specific traumagenic dynamics: powerlessness, betrayal, stigmatization, and traumatic sexualization. Given the severity and consequences of sexual assault, it is essential that child victims of abuse receive prompt treatment (Centro de Asistencia a Víctimas de Atentados Sexuales [CAVAS], 2011). Specialized treatment options have been proposed in order to address the complexity of this problem. An eco-systemic approach, for example, brings together different therapeutic actions aimed at reestablishing overall functioning of children, allowing them to elaborate the traumatic experience (Gómez et al., 2010; Centro de Asistencia a Víctimas de Atentados Sexuales [CAVAS], 2011), being this kind of psychotherapy one of the ways of treatment.

In psychotherapy with children who have been sexually assaulted, therapeutic approaches must constantly adapt and adjust to the patient’s own rhythm—incorporating and reaffirming his or her needs while also adhering to the goals of treatment (Cattanach, 2004). Inside this, play is regarded as a key therapeutic activity for children because it stimulates communication and the symbolization of lived experiences using non-verbal methods of expression. This is especially important when working with victims of sexual assault who may find it difficult to speak about their experiences (Gil, 2006; Esquivel y Ancona et al., 2007). Play also supports general development and creativity in children (West, 2000; Cattanach, 2004); it provides a safe and protected space where children may experiment with different ways of acting and understanding the world and articulate and integrate traumatic experiences (West, 2000; Oaklander, 2001).

Symbolic play is a form of expression in which the comprehension of self and reality are transformed using symbolic language (Piaget and Inhendler, 2000), enabling children to return to past experiences and take control over them (Piaget, 2010). In this way, a child may act out traumatic experiences using repetition compulsion of an event in order to assimilate their experience—referred to as post-traumatic play (Gil, 2006). Aspects of trauma are acted out in a more direct and less imaginative way than in typical child’s play, and involve the repetition of a specific troubling outcome (Terr, 1990). Nonetheless, as children take control over how they relive a traumatic experience in a safe and contained environment they may then externalize the event, moving from passive receptor to active agent (Ogawa, 2004). As a result, they are able to develop their own account of the experience and create new meaning (Freeman et al., 1997).

Sand tray therapy, a symbolic mode of play, is commonly applied within clinical settings. Here, children use a sand tray to create their own fantasy worlds. Through the use of different figurines (miniature animals, people and plants, among other items), children depict their understanding of the world and aspects of their reality (Amman, 1991; Dale and Lyddon, 2000), providing the clinician with a symbolic representation of their inner world (Cunningham and Macfarlane, 1996). This playful approach provides children with a contained and protected space for where they may share facets of their experience (Labovitz and Goodwin, 2000).

During the sandplay it is important to pay attention to both behaviors as well as the content of the game, which provides a deeper understanding of the activity for further analysis an therapeutic use. Such observations may include how children handle sand, the number of miniatures utilized, the ways in which children engage in play, as well as any changes or revisions made to the game. Content refers to emerging themes played out in the fantasy world and any connections between them (Sjolund and Schaefer, 1994).

In this way, a number of authors have shown the benefits of sand tray therapy, or sandplay, in young patients’ progress during psychotherapeutic treatment (O’Connor and Schaefer, 1994; Oaklander, 2001). In particular, research on the use of sandplay with child victims of sexual assault (Grubbs, 1994; Dripchak, 2007) reveals that by using sandplay children may externalize conflict and take control over negative experiences, opening the possibility to portray, manipulate, alter and destroy facets of the traumatic experience. These are all crucial to psychotherapeutic work in the sense that they allow individuals to resignify events (Labovitz and Goodwin, 2000).

On the other hand’s the effectiveness of different forms of play therapy for child victims of sexual abuse has been studied (Hetzel-Riggin et al., 2007; Sanchez-Meca et al., 2011). Nonetheless, research often fails to describe the characteristics of these activities during different stages of treatment (Mathis, 2001). Moreover, despite a surge in international research on the use of play during therapy (Harper, 1991; Zinni, 1997; Homeyer and Landreth, 1998), researchers often comparing play in children to other clinical samples or control groups, allowing the analysis of differential indicators, such as sexuality, safety, and care, that emerge in play. Despite the relevance of these research findings, indicators are isolated from the larger context of psychotherapy, in which sand tray therapy is limited to evaluating a specific moment of the therapeutic process.

Few studies employ methodologies that allow for a deeper analysis of aspects of play therapy and their progression over the course of psychotherapy (Mathis, 2001; Herane, 2005). In one case, Mathis (2001) analyzes the progression of sand tray therapy among children during 10 months of treatment. Results show a positive change in how children engage in sand play.

Considering all these, limited research on the topic of therapeutic treatment with victims of sexual assault, particularly play therapy and children, demonstrates the need for further qualitative studies concerned with the subjectivity of participants involved in treatment. The present study therefore seeks to analyze characteristics of play during therapy among child victims of sexual assault between the ages of 7 and 10. Three particular moments of the therapeutic process are observed using sand tray therapy.

## Methodology

### Sample

The research sample consists of children between 7 and 10 years old. Participants were selected using inclusion criteria of both girls and boys who have suffered from sexual abuse and who, at the time of study, were enrolled in psychotherapeutic treatment in a specialized center for the treatment of child sexual assault. The sample was obtained at the Center for the Treatment of Sexual Abuse (CAVAS), an organization affiliated with the Policía de Investigaciones de Chile (Civil Police Department) which serves child victims of sexual assault in the city of Santiago, Chile.

In order to obtain and establish the sample, authorization was secured by the Institute of Criminology which oversees CAVAS, specifically by the project team coordinator which provided researchers with access to clinical records of the children who agreed to participate in the study.

Therapists were asked to present the option of participating in the study to patients and their legal guardians. They were then informed of the research study, as well as ethical implications of participating voluntarily and anonymously. After agreeing to participate, the parents or legal guardians were asked to sign an informed consent form, and children’s verbal assent was obtained by the therapist once they confirmed their desire to participate. The total number of participants was determined using the criterion of data saturation (Rodríguez et al., 1999), which considers participants until all information has been sufficiently collected. Cases of sexual abuse among the sample population were either extra- or intrafamilial and differ in terms of incidence and duration, particularly in terms of chronic and repeated exposure. This criteria is consistent with literature on sexual assault and the cases share similar characteristics (Finkelhor, 1994; Cantón and Cortés, 2004; Pereda et al., 2009). In addition, differential criteria were included, specifically regarding gender and participants with varied symptomatology. It is important to note that cases involving serious psychological disorders, such as psychosis and organicity, were omitted from the sample. Accordingly, the primary characteristics of the sample population are presented in **Table 1**.

**Table 1:** Sample characteristics.

Characteristics	Category	Number
Gender	Girl	5
	Boy	2
Relation to perpetrator	Paternal figure (father, stepfather)	2
	Other family member (brother, grandfather, uncle, great uncle)	3
	Acquaintance (family friend, other)	2
Type of aggression	Sexual abuse	3
	Rape	4
Incidence	Repeated	5
	Chronic	2
Primary symptomatology (could be more than one)	Depressive	5


	Anxious	7
	Behavioral	4
	Somatic	2


All children participating in the study had completed psychodiagnostic phase, which identifies clinical indicators of sexual abuse prior to starting treatment, and were in the beginning stages of psychotherapy. Participants attended sessions on a regular basis and, according to information reported by therapists for each case, children had no contact with the perpetrators.

With regards to the treatment method, sand tray therapy was used prior to the study in order to familiarize participants with the activity, but wasn’t the only technique used in therapy. It is important to note that the type of sand tray and miniatures utilized in the present study are consistent with the technical references established by specialists (Cunningham and Macfarlane, 1996; Gil, 2006).

On the other hand, about the sample of therapists, one selection criteria in the present study was that therapist uses implementation of a constructivist approach to psychotherapy. Therapists were also expected to have had previous experience or prior training with sand tray therapy in a clinical setting. This was important for standardizing the application of techniques and interventions employed in the study. Finally, a total of six psychologists participated in the study, all of whom took part in a theoretical and practical training course on standardizing criteria related to the application and engagement with sand tray therapy.

It is relevant to note that the present study was an investigation carried out in the context of a Master of Clinical Psychology Program at the University of Chile (Tornero, 2014). Thus, the ethical aspects of this research, along with other aspects, was fully assessed, reviewed, and approved by a specialized review committee at this Program, which uses guidelines of the Ethics Committee for the Investigation in Social Sciences and Humanities of the University of Chile.

### Techniques

One of the main methods for collecting qualitative data was observation. This involves the detailed and systematic description of behaviors, artifacts, and events in the social setting under study (Marshall and Rossman, 1989; Banister et al., 2004) in order to record both verbal and non-verbal behaviors of the subject in his or her specific context (Banister et al., 2004). Observation, when paired with play therapy, provides insight into this phenomenon because it looks specifically at narrative and behavioral components of creative play. This is consistent with the key objectives of the present study.

Observation is beneficial in cases where subjects have difficulty expressing themselves verbally and emotionally, thereby allowing researchers to access a variety of topics, unlike other techniques (Rodríguez et al., 1999). It is a minimally invasive method of study for this specific sample population, which requires an approach that is attentive to the psychological impact of sexual assault and protects the psychosocial well-being of participants.

Moreover, this technique draws on recording methods that help to structure and guide the process and specify the content and length of time under observation (Banister et al., 2004). In the present study, observation was structured using two primary methods: video recordings and an observation rubric or assessment.

Prior to beginning, a rubric was developed in order to provide a framework with which to observe and evaluate behaviors, content of the activity and the therapeutic relationship. A theoretical review of the literature on sand tray therapy was conducted and relevant aspects of the activity and the relationship between therapist and patient were identified and systematized in the rubric. Behaviors and content of sandplay served as a primary focus for understanding the various activities and narratives developed during the activity. With regards to the therapeutic relationship, verbal and non-verbal interactions, as well as displays of emotion and exchanges between patient and therapist, were considered. This was a way to essentially grasp the relational context within which sandplay was developed, providing an additional perspective in order to advance understanding of this therapeutic tool. Within each of these three aspects (content, behavior, and relationship), more precise aspects of observation were identified. Nonetheless, for the purpose of this article, only behaviors and content of sandplay are explored.

It is important to note that the observation rubric was reviewed by a professional psychologist specialized in psychotherapy with child victims of sexual abuse, and all suggestions were incorporated. Similarly, the rubric was applied to a pilot case independent of this study, allowing researchers to make changes prior to implementation. This rubric has the objective of guiding observation (for example, to observe use of sand, story development, etc.) and not to code behaviors.

In the second phase of study, therapeutic sessions for seven children receiving treatment at CAVAS were videotaped. Video recordings are essential for analyzing the actions and interactions of participants, and they allow researchers to observe a situation more than once (Secrist et al., 2002). Moreover, a photo digital camera was used to obtain a visual image of the sand tray and the final product. Images are not analyzed in the present study, however, they are used as an example.

To adhere to the normal course of treatment for ethical reasons, children’s own therapists were therefore responsible for introducing the activity into their therapeutic sessions. Therapists presented each child with material components of the play and then provided them with the following instructions (based on guidelines by O’Connor and Schaefer, 1994; Homeyer and Sweeney, 2011): “Create your own world or story in the sand, exactly how you would like to, using the miniatures provided. Once you have finished, we will take a picture of the sand tray.” The materials used were a plastic container. According to the guidelines provided by authors such as Mathis (2001), they were ideally, blue so that when sand is shifted within the box it gives the impression of water or the sky (Mathis, 2001). Moreover, the recommended size of the box was used, which is 46 cm × 69 cm with a 5 cm border allowing for easy observation of the activity (Lowenfeld, 1979; Oaklander, 2001).

Video Recordings were taken at three different points in time during the psychotherapeutic process: in the beginning, after 3 months of treatment and finally, after 6 months of treatment. This allowed for a span of six to nine regular sessions between each activity, which complied with long-term treatment goals while also minimizing the number of sessions between recordings. The timeline was implemented at the start of treatment, as clinical manifestations of the activity in the beginning stages of treatment were shown to provide key information on the progression and psycho-affective state of each child. At the same time, the activity was recorded during the initial session and in two other points of treatment in order to gauge the activity’s progression (see **Table 2**).

**Table 2:** Timeline of video recordings.

Participant	Sex	Age	Number of recordings during the initial phase	Number of sessions from beginning to month 3 of treatment	Number of recordings at month 3 of treatment	Number of sessions in months 3–6 of treatment	Number of recordings at month 6 of treatment
M	F	10	1	6	1	6	1
I	F	8	1	8	1	7	1
J	F	9	1	7	1	8	1
F	F	8	1	8	1	-	0
W	F	7	1	8	1	8	1
V	M	8	1	6	1	7	1
B	M	8	1	9	1	-	0


It should be noted that during the research process, some cases withdrawn from the study. As a result, there are seven recordings from the first phase, seven from the second and five from the third and final phase.

Recordings were scheduled together with the therapist, informing to the child and legal guardians of this prior to the session. Practice recordings were performed in order to ensure proper positioning of the camera without interfering with the session. In between recordings, participant’s progress (attendance and general emotional state) was monitored by the therapist as to determine the best time to schedule a future video recording of the sandplay session. In this way, the researcher was able to access relevant information about the therapeutic process and the child’s own history, and to gain a deeper understanding of the particular case.

Therapeutic sessions were approximately 50 min long, and the duration of the activity itself ranged anywhere from a fraction of the time allotted to the full session. Recordings were taken of the entire session, regardless of the time spent on the activity, in order to ensure the normal order of the psychotherapeutic session. The sand tray was photographed prior to disassembly. Finally, observations were made by researchers using recordings of therapeutic sessions, the transcription of the videos and the observation rubric. All steps of the process were repeated for each phase.

### Analysis

Data gathered through observation was analyzed using qualitative content analysis, one research method concerned with subjective interpretation. This is important given the present study’s focus on play as a form of expression among children. Content analysis is used for textual material produced by a number of data gathering methods, thereby allowing the researcher to access content of any communicative act. It also outlines a set of procedures for organizing both verbal and behavioral information (Abela, 1998; Pérez Serrano, 1998).

Content analysis was employed using guidelines proposed by Strauss and Corbin (1990). This method involves selecting relevant parts of a text and identifying primary units of meaning which are related to phenomena under study. These units of meaning are then qualitatively coded and categorized using specific criteria (shared characteristics and meanings) in order to later develop an interpretive understanding of data, making comparisons of theoretical information while further developing the research question (Strauss and Corbin, 1990; Hernández et al., 2006).

In the present study, videos and their transcriptions were observed using the rubric. Given the variety of data sources in terms of both digital and analog language, it was key to develop a form of qualitative coding which would allow the research to carry out a detailed analysis and consistently work with information in order to clarify observations. Coding was done identifying portions of the text, that were selected and grouped according to thematic criteria presented in the rubric (for example, identifying feelings in the play, which were of anger, fear, etc.). These were directly related to the objectives of the present study, and some were eventually designated as categories.

In the second stage, a cross-sectional analysis was carried out for each phase of sand tray therapy. Results led to the development of a conceptual map for interpreting content, and a document, or memo, was developed for each of the phases. In the third and final stage, and once the conceptual map was created for phase, characteristics of categories present in the transcriptions were examined and compared. Categories were evaluated and reformulated based on emerging meanings in order to identify similarities and differences between phases. It is important to note that two researchers were involved in reviewing recordings and defining categories, using intersubjectivity in order to verify observations and analysis, presenting generally, important similarities in the analysis. Thus, the triangulation of data analysis among researchers was a significant criteria of rigor in this study.

## Results

Results are organized according to the behaviors and content of sandplay observed in psychotherapeutic sessions. In the first section of each content or behavior, commonly observed aspects which appear at different phases of treatment are presented. Differences between the three phases are then identified. Main results are shown in **Tables 3**, **4**. Excerpts from video recordings as well as photographs of the sand tray help to illustrate aspects described in the categories of analysis and provide concrete examples of elements described in the paper, while respecting the confidentiality of all participants at all times.^1^

**Table 3:** Main results of play behavior.

	Play Behavior		
	
Aspects of sandplay	Phase 1: Beginning of treatment	Phase 2: After 3 months of treatment	Phase 3: After 6 months of treatment
Use of sand	Sustained contact with the sand: Sensorial activity/Anxious when explaining conflicts. Behavioral characteristics: Shaping-pouring-burying-digging. Sand plays an important role in creating the backdrop of the story.		Sustained contact with the sand, which intensifies when child is in distress.

Categories employed in the activity	People: Family members, police, soldiers and/or warriors.	Similar to those found in phase 1. Gradual inclusion of reptiles, especially snakes, who appear to be menacing and dangerous.	
	Fantasy characters: Superheroes, bad guys, ghosts, and monsters.		
	Animals: wild and domesticated		

Characteristics of the activity	Prevalence of symbolic play	Symbolic play Predominance of mobile play	
	Predominance of rigid play		

Use of time	Predominantly spent building the fantasy world, followed by story development.		Sporadic interruptions during the game.
	Delays and interruptions associated with content of the game.		

Composition	Two major types: Meaningful and consistent scenarios; Increasingly chaotic and disorganized.		Worlds that are predominantly composed in a meaningful and coherent manner.


**Table 4:** Main results of play content.

	Play content		
	
Aspects of sandplay	Phase 1: Beginning of treatment	Phase 2: After 3 months of treatment	Phase 3: After 6 months of treatment
Emerging topics	Violence	Violence	Changes in central themes of participants’ stories: Reduced widespread violence, which becomes more specific to certain interactions or behaviors. External support when encountering physical danger.

Character identity	Central focus: good and evil; good characters protect and provide support to others when faced with dangerous situations or aggressors, providing direct assistance; bad characters: unlawful behaviors.		

Surroundings and environment	Hostile and dangerous: threatening and/or threats to public order, between animals, humans or natural disasters, which put all characters at risk.		More organized and less destructive. Presence of safe zones where characters may seek refuge and access personal resources.

Needs	Need for protection and care from threats in one’s immediate environment.		Needs are generally met as a result of one’s own personal resources or the actions of others.

Feelings and emotional atmosphere	Predominantly marked by fear, which all characters experience in specific contexts or interactions.	Predominantly marked by fear, which all characters experience in specific contexts and interactions; however, progressive inclusion of positive feelings (affection, hapiness, trust).	

Traumagenic dynamics	Predominantly marked by feelings of defenselessness and fear associated with not having control over harmful external events.	Predominantly marked by feelings of defenselessness and fear associated with not having control over harmful external events. Also other dynamics are present: betrayal, traumatic sexualization and stigmatization.	

Story development	Imaginative outcomes with atypical and eccentric or ambiguous features.		Predominantly happy endings, where threats are brought under control and protagonists find a safe space.


### Play Behavior

#### Use of Sand

Sand plays an important role in all three phases of treatment, particularly in the development of a story’s setting. Four characteristic behaviors were observed among participants: shaping, pouring, burying or hiding, and digging.

Participants initially shape sand in order to even out the surface of the sand tray, which they can then use to construct elements resembling their natural environment or structures, such as mounds or hills. The act of pouring sand is primarily a sensory activity (especially in children with risk-taking behaviors), and registers different levels of intensity depending on whether sand is moved with force or lightly sprinkled. Participants may bury objects or parts of the body (hands or fingers) partially or completely only to later reveal them. Finally, digging in the sand allows participants to create small furrows in the sand with their hands or objects, which serve as waterways (lagoons, rivers, the ocean), hiding or storage places or spaces to bury fallen soldiers.

During the more advanced phases of treatment, changes in behavior include prolonged contact with objects, which grows in intensity when participants expresses sadness over one aspect of game. This may manifest, for example, in an altercation between two people within the game who threaten or hurt each other or exhibit distress.

#### Categories Employed in the Activity

In order to create their own worlds out of sand, participants drew on human miniatures, fantasy characters and animals of different sizes and species with varied identities, abilities, and duties. Moreover, one important observation was the repeated use of certain categories in all three phases of treatment.

Humans employed in the activity were often family members (grandparents, men, women, teenagers, and young children), public servants (police) and service members (soldiers, warriors, or fighters). Fantasy characters were mostly male and rarely female. While heterogeneous in form, fantasy characters generally possessed special powers and were engaged in fighting. In particular, superheroes and magicians had a dominating presence and were able to exercise control over others.

With regards to the use of animals, participants employed both wild and domestic creatures in families, with each individual carrying out a particular function. From the second phase onward, many participants began to employ reptiles, particularly snakes—a threatening and dangerous element of the game. In the same vein, the incorporation of mythological creatures such as dragons, which play a prominent role in participants’ stories, is suggestive as they represent both power and evil.

#### Characteristics of the Activity

**FIGURE 1:**
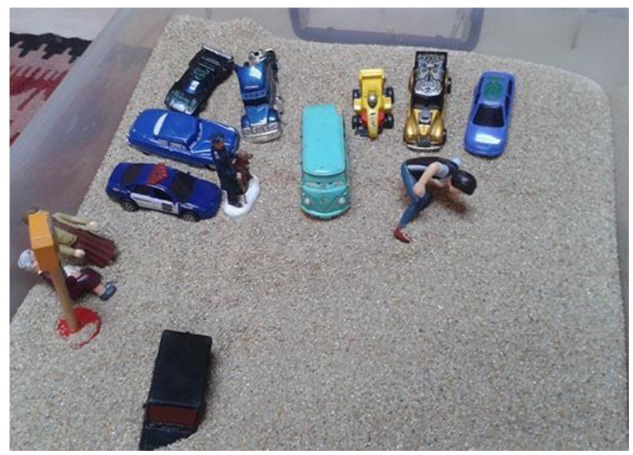
**Example of rigid play (I-V2)**.

Generally, throughout the treatment process, participants developed a symbolic game involving complex story lines that integrate aspects of reality and fantasy. Here, there are two primary modes of playing. The first involves selecting and arranging figures within the sand tray until a scene is completed. These figures remain in a fixed position throughout the game (see **Figure 1**).

**FIGURE 2:**
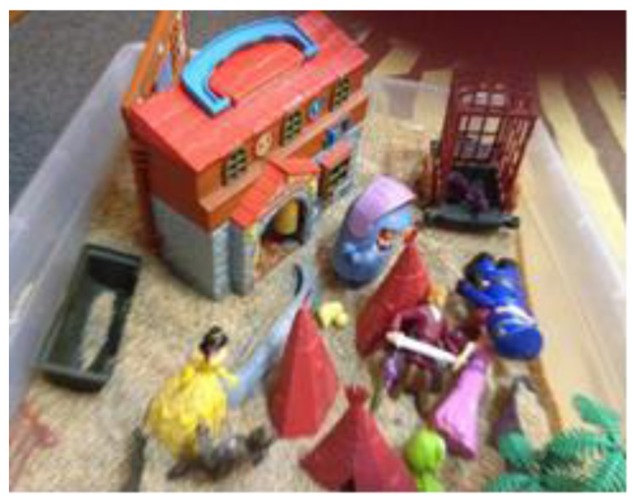
**Example of mobile play (W-V2)**.

The second mode involves movement or action, whereby the development of a story line is coupled with movements. Here, scenes are active and accompanied by speech, emotional responses, and sounds (see **Figure 2**).

In the initial phase of treatment, participants demonstrated stagnant play, whereby objects are arranged in a static manner despite changes in the story. This contrasts with the later stages of treatment, which were dominated by dynamic play, whereby miniatures and their respective personalities interact with each other and move in different directions, even extending beyond the limits of the sand tray with the development of the story.

#### Use of Time

Participants dedicated a majority of their time to constructing physical aspects of the play world and then spent the remaining time developing a storyline. These moments were clearly demarcated and communicated to the therapists. In addition, the presence of delays or interruptions is one important aspect of sandplay and was a central component observed among participants in this study. This is associated with a number of interrelated factors, such as content of the game and moments of introspection in response to therapeutic interventions.

Interruptions during play were common and particularly recurrent in the first two phases of treatment, while in the third phase, interruptions were more sporadic. The following excerpt shows the relationship between a desire to pause the game and one’s own biological functions. Here, the child expresses her need to use the restroom and physically remove herself from the space. This may be related to emerging feelings of anxiety based on the progression of the game: The child turns toward the box, searching for new objects and says: N: “I have to go to the bathroom” –using a quiet voice (V-V1).

#### Composition

With regards to the composition of the sand tray, participants organized their worlds in two ways: first, developing meaningful and consistent scenarios where miniatures and other elements in the sand tray are directly implicated in the child’s story and second, progressively creating a scenario that becomes more chaotic, disorganized and filled with toys. In the second case, stories began to lose their logical sequence, making them difficult to understand. This is shown by the following excerpt, or observation of this type of disorganization:

T: “Ok, what is the story of this world you’ve created?” N: “it has to do with, um, with a birthday” N: “both of their birthdays” – she takes up the princess and moves its closer to the prince, then turns to face the sand tray N: “Ah!” – she says, taking the duck out of the sand and quickly placing it on the edge of the sandbox, which represents the ocean – N: “the duck was leaving, I don’t know” (W-V3).

In contrast, phase three of treatment is generally marked by more composed scenarios and coherent and meaningful worlds.

### Content

#### Emerging Topics

Among the many topics that emerged during sandplay, violence served as a key organizing theme. Here, content related to experiences of victimization referred to both individuals and groups of people who act in ways that cause physical, psychological, and social harm. Violence in its various forms serves as a guide for the stories that participants develop throughout the course of treatment, whether it be widespread or an individual act that threatens the welfare of others.

During these scenes of aggression, the position of victim and perpetrator are clearly defined, sometimes shifting as the child’s story develops. The perpetrator appears as a powerful and all-knowing figure who uses a variety of strategies in order to subdue the victim, using trust as a manipulation tactic, as well as physical harm, deceit, and persecution. The victim, however, suffers from physical harm by others, feeling defenseless and fearful as a result.

**FIGURE 3:**
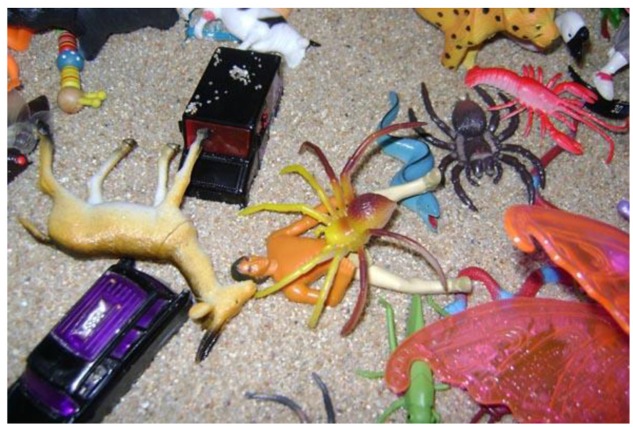
**Example of violence in play (V-V1)**.

With regards to the wide range of negative interactions observed during sandplay, participants referred to three specific types of asymmetrical relationships: aggressive, abuse, and degrading. These relationships are interrelated and marked by an attack on part of the perpetrator, who employs both verbal and physical violence such as killing, hitting, and biting. This is demonstrated in **Figure 3**, where one participant develops a scenario in which an aggressor (characterized as a spider) kills a man by the venom of its bite.

During play sessions, participants’ stories often allude to serious offenses toward female figures or between male figures and others who are typically the most vulnerable due to their size, gender (men–women), stage of development (children–adults) and category (animals–humans). For example, the following excerpt provides insight into the aggressive interaction between a father and his daughter, involving both physical and psychological violence: N: “And she started to cry” T: “mmm” N: “honey, don’t cry” –sound of hitting N: “don’t cry” – another hit – N: “don’t cry, he was hitting her” T: “And why?” N: “because he didn’t want her to cry (…) you’re stupid” – sounds of fighting (F-V2).

The third phase of treatment shows marked changes in the ways in which violence is presented, mainly the shift from widespread violence to particular violent interactions or behaviors. In some cases, miniatures that were previously used to attack or defend against others do not engage in violence. In addition, stories feature more positive interactions involving collaboration, care and safety. These are often intended to protect characters from violence or to provide support to characters that have been hurt.

Additional emerging themes in participants’ stories include threatening environments (hostile and dangerous surroundings), safety measures (to defend against threats or danger), social control (authority and public order), transformation (characters that shift from bad to good or that die and are reborn), family (dynamics and social roles) and nourishment (feeling full or hungry).

#### Character Identity

In general, throughout all phases of treatment, participants’ stories involve plot lines where evil and good are central to identifying characters in the game. On the one hand, “good” individuals are characterized by their ability to protect and provide aid to others in the face of aggressors or dangerous situations. For example, a policeman reports and imprisons those who commit crimes. On the other hand, “bad” individuals are identified by their unlawful behaviors and violent actions toward others, such as physical attacks, theft, destruction of property and harassment.

#### Environmental Conditions and Personal Needs

Environmental conditions present in the participants’ stories are typically dangerous and hostile, and involve various forms of threat and aggression on part of animals, humans, and natural disasters. In particular, tornados and tsunamis appear to create chaos and destruction, resulting in death and material losses and negatively impacting the community. The following excerpt provides an example of the destruction and fatalities caused by a natural disaster: N: “I am searching for survivors!” T: Survivors from what? “N: The tsunami” T: Ahh N: Instead of water there was only sand and all the sand swelled up” T: And the monkeys that were there (…) N: “buried” (V–V3).

In the third phase of treatment, stories draw on environmental conditions that while still threatening and harmful, are noticeably more organized and less destructive than in previous phases. In addition, there is the presence of safe zones, where characters may take shelter or access personal resources that allow them to continue on with their life.

With regards to personal needs, children refer to a lack of protection and care in their stories and draw on feelings of insecurity in the wake of environmental disaster. Here, individuals within their immediate circle (family or friends), as well as law enforcement, play an important role. Moreover, during the third phase of treatment, children’s stories involve characters whose needs are met either by themselves or by others. For example, a character’s need for protection is met with a safe space to stay, or a character is rescued from danger.

#### Feelings and Emotional Atmosphere

Participants’ stories developed through sandplay at different stages of the therapeutic process are dominated by feelings of fear. Here, characters experience fear when they come into contact with external, hurtful events that are out of their control. As a result, the characters develop a number of psycho-affective and physical responses which vary, even including loss of consciousness and fainting:

N: “she got scared because a girl was walking by with her grandmother” T: “and what happened with the girl and her grandmother?” N: “they fainted” T: they fainted! N: “yes, because the car drove by really fast” T: “mmm, so they got scared.” N: “Yes” (I–V2).

Furthermore, feelings that emerge while playing include anger, confusion, sadness, self-reproach, shame, loneliness, hopelessness, and anxiety, all of which refer to catastrophic thinking. Physical feelings of illness and pain related to bodily injury are also present. With the progression of therapeutic treatment, participants’ stories involve more circumscribed, positive feelings associated with particular interactions, most notably affection, happiness and trust, which are grounded by an emotional connection to their environment.

In terms of the consequences of sexual assault, or traumagenic dynamics articulated by Finkelhor and Browne (1985), feelings of defenselessness and fear are present in participants’ stories. These are often associated with a lack of control over harmful external events. In some cases, characters in the story struggle to remain safe and protected. Despite their search to find a place to hide, threats from their immediate environment are overwhelming: T: “some are trying to steal, others want to raze the building” N: Over there, the girls are hiding (…) N: “There are bad people everywhere” (V–V2).

On the one hand, betrayal appears as a theme in the first and second phases of treatment. Participants’ stories speak to a feeling of mistrust between individuals, which is characterized by low credibility, hostility and aggression and which acts as survival mechanisms (fight or flight) in their interactions.

On the other hand, traumatic sexualization is observed in phases two and three of treatment in some participants’ stories. This involves an eroticized interpersonal relationship between figures of the same or opposite sex. Some children who suffered from sexual assault at the hands of someone in their immediate family acted out abusive and humiliating moments between characters. In these cases, individuals were treated as commodities or objects to be exchanged and were seduced (particularly through gift-giving) by another individual. This is reflected in the following excerpt:

N: “I’ll give you my little duck from ule if you dance with me” T: (laughs) “but you already told him that you would go to the dance with him” N: (laughs) “I’ll give you, I’ll give you to my girl cousin” T: “How are you going to give me to your cousin?” N: “I’ll give you to her” T: “You can’t give me to another person” N: “I’ll give you to, I’ll give you to my boy cousin” (W-V2).

In phase one, stigmatization is present only in the cases of children who have suffered from sexual assault committed by individuals outside of the family (acquaintances). Throughout the course of therapy, however, an increasing number of participants (the majority of children) allude to stigma in their stories in phases two and three. Contents of the play session include characters that are isolated and have feelings of inadequacy, shame, and social exclusion. For example, one child says *N*: “*it feels bad because it’s different from all the other animals*” *(M-V1).*

#### Story Development

**FIGURE 4:**
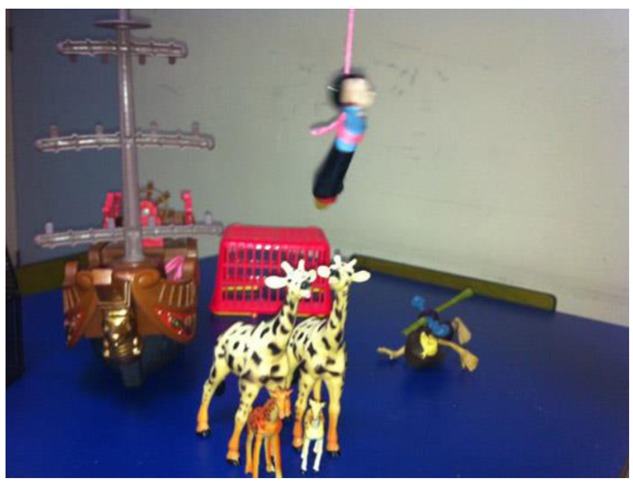
**Example of fantasy in stories (F-V2)**.

In the majority of cases, participants create a fantasy world with atypical and eccentric features. Accompanying stories are often ambiguous and incomplete, ultimately lacking an end or a final close. For example, **Figure 4** illustrates one imaginative turn in the story where a flying child appears, without any relation to the prior storyline.

In phase three of treatment, changes in story development were positive in most of the participants: threats were taken under control and protagonists of the stories were able to find a safe space. At the same time, the development of support networks among characters signaled a shift in the ways in which characters behaved. Originally faced with social isolation, characters received help by others in order to move elsewhere or reach their own goals.

## Conclusion

The present study explores the topic of creative play in therapy with child victims of sexual assault. In particular, it examines their therapeutic progress through the use of sandplay, or sand tray therapy, using a qualitative approach. These allows us to explore how children narrate and rewrite their lived experiences and how therapists serve as co-participants in the process of metaphorical reconstruction (Dale and Lyddon, 2000). Key findings show a common set of behaviors during play that are related to violence: characters involved in the game act in an aggressive manner, they require care and safety and act out imaginative endings to conflict. Moreover, there are marked differences in the different stages of treatment, showing a gradual progression toward the third stage, where the activity involved mobile play and the creation of meaningful worlds with more organized environments and an overall positive outcome.

Results are consistent with international studies on the subject (Cunningham and Macfarlane, 1996; Zinni, 1997; Homeyer and Landreth, 1998). Specifically, the use of digging or hiding objects while playing with sand has been widely observed among children who have suffered from sexual abuse, which serves as a metaphorical representation of the secrecy surrounding such experiences (Cunningham and Macfarlane, 1996; Homeyer and Landreth, 1998). Nevertheless, it is important to analyze the specific meanings assigned by each child.

Creative materials employed during sandplay include people, fantasy figures and animals, all of which have been described by other authors during the use of therapy with child victims of sexual assault, specifically wild and domesticated animals, boyfriends, magicians, and superheroes (Cunningham and Macfarlane, 1996). In the present study, participants predominantly referred to male figures in their games, which may refer to an association between masculinity and authority or power. This is one aspect that may be explored in future studies.

One of the most relevant miniatures employed during the activity is the snake, which was progressively incorporated in child’s narratives over the course of treatment. This has been detailed in previous studies on the symbolism of animals in therapeutic treatment of child victims of sexual assault (Cunningham and Macfarlane, 1996). During play sessions, snakes carry the connotation of causing harm and generating fear, and they are incorporated and taken away throughout the game. From an analytical perspective, the snake may be a phallic symbol over which the individual assumes control (Bettelheim, 2010). On this basis, and considering the presence of the snake as a prominent character in the third phase of therapeutic treatment under study, we may speculate that children begin to symbolically address sexualized aspects and feelings associated with experiences of abuse. Here, disassociated memories may emerge through metaphors employed during play once the child feels that the therapeutic space is safe and contained (Colombo and Beigbeder, 2005).

The composition of the game, which gradually becomes less chaotic over time, is similar to other experiences in past research on the presence of disorganization in sandplay with victims of sexual assault (Zinni, 1997). In these cases, the development of chaotic worlds reveals distortions in how children perceive their reality during an emotional crisis caused by a traumatic experience – a topic that would benefit from further analysis (Sjolund and Schaefer, 1994). In the present study, it is important to note that the composition of sandplay becomes more organized as treatment progresses.

Additional behaviors present during sandplay include sustained contact with the sand, particularly during the advanced phases of treatment. When children become distressed, handling sand may serve as a control mechanism for their emotions as they act out conflicts in the game. Many authors have regarded sandplay as a way to provide children with a sensorial experience that has therapeutic benefits by promoting relief and relaxation (Oaklander, 2001; Gil, 2006). Results of the present study as well as past research may speak about the important role that sand plays in the therapeutic process of children who have suffered from sexual assault. Sand provides them with a contrasting tactile experience where they may reconnect with their own body and experience positive and pleasurable sensations that promote physical and psychological wellbeing. Moreover, this activity allows children to freely build and destroy their own creations, providing them with a sense of control that was taken away from them as a result of sexual abuse (Gil, 2006). Finally, the therapist may serve as both an observer and supportive figure, guiding the child in his or her own search for meaning.

With regards to game or play content, the topic of violence plays a central role in participants’ stories. This has been also shown by previous studies (Zinni, 1997; Herane, 2005; Sandoval, 2005). Moreover, these findings are consistent with other studies using projective techniques, specifically Rorschach, which suggest that violence plays a central role for sexual abuse victims, affecting their inner world (Armstrong and Loewenstein, 1990; Kamphuis et al., 2008; Scimeca et al., 2015). These studies show that victims of sexual abuse tend to experience intrusion of traumatic memories into consciousness, as well as a negative perception of the world. They also tend to show a more disorganized perception of causality in the understanding of human relationships (Armstrong and Loewenstein, 1990; Kamphuis et al., 2008; Scimeca et al., 2015). Thus, it is possible to think that both, sandplay and projective techniques for personality assessment, share the common characteristic of allowing individuals to freely reproduce their inner world.

Similarly, the worlds that children create during sandplay invoke both fear and distress; the emotional atmosphere is reflected in content of the game, which reveals a traumagenic dynamic of defenselessness associated with experiences of sexual abuse (Finkelhor and Browne, 1985). As a result, children draw on the need for protection and care in their stories, and authority figures such as law enforcement (police) play a vital role, which is consistent with other research studies (Homeyer and Landreth, 1998).

Moreover, when taken together, results of the present study on behaviors and content are consistent with those presented in the literature on post-traumatic play—a ludic form of therapy observed with child victims of sexual assault (Terr, 1990; Cunningham and Macfarlane, 1996; Gil, 2006, among others). In this way, it is clear that traumatic events associated with violence are reproduced during treatment in terms of the level of engagement with certain topics and play objects, as well as stories associated with feelings of risk and threat, or more generally, an emotional state of fear and defenselessness. Unpleasant emotions experienced during the game, such as anxiety, are accompanied by analog movements such as delays, silences, and interruptions. These findings are consistent with clinical descriptions of post-traumatic play, and they underscore the value of monitoring and providing follow-up to ludic processes in a rigorous and systematic way (Terr, 1990) in order to promote approaches that respond to the changing needs of children.

At the same time, differences across categories in all three stages of the process provide insight into the evolution or progression of post-traumatic play over the course of psychotherapeutic treatment for child victims of sexual assault. Content of sandplay reveals a shift in terms of violence; in later phases, worlds developed during sandplay feature safer and more organized environments, as well as individuals who have the capacity to confront threats in their environment and arrive to a positive conclusion. Behavioral aspects of the game include mobile play and the development of meaningful and coherent worlds. Fewer disruptions demonstrate a more integrated and less fragmented game.

These results are consistent with previous studies by scholars such as Gil (2006), who argue that the transition from stagnant to dynamic play is representative of a child’s ability to slowly integrate emotions and thoughts affiliated with a traumatic experience and assign meaning to and assimilate the experience. While conflict and unpleasant emotions may still be present despite these transformations, the progression of post-traumatic play is clear. The game becomes more dynamic and encourages the child to approach the topic in a symbolic manner, empowering and providing him or her with an emotional release.

In general, while children may experience the pain and consequences of sexual abuse in different ways, study results show that there are certain common ludic expressions among child victims, which are demonstrated through sandplay at various points in time during psychotherapy. These expressions are consistent with findings from previous studies, and are representative of the primary challenges faced by this population. In addition, there are certain characteristics of sandplay which evolve over the course of psychotherapy, and which demonstrate key changes in play that may support children in redefining and assigning new meaning to their experiences.

The present study contributes to the literature by providing new insights on the application of sandplay as a therapeutic tool for child victims of sexual assault, opening up new lines of work on the process of overcoming abuse. In this sense, it is a major contribution to research which foregrounds children’s perspectives and means of expression.

Findings of the present study reaffirm the value of therapeutic play in cases of sexual abuse, which can provide rich insight into the psychological resources of children that help to facilitate a positive emotional change. In this way, it may help the therapist to delve into the meanings that children develop and assign to their experiences and monitor their engagement with symbolic elaboration. Since the present study follows participants during 6 months of treatment and not necessarily to completion, future studies are needed to explore the evolution of creative play in terms of the entire psychotherapeutic process. Additional studies may benefit from a comparative analysis of the characteristics of sandplay in both the initial and concluding phases of treatment.

In methodological terms, the present study employs a longitudinal research approach to therapeutic treatment. Here, the researcher had direct access to medical records. It presents a focus the evolution of creative play and the value of exploring changes in play throughout the course of treatment, which is a research design that may be explored in more detail in future study. By conducting a general analysis of heterogeneous cases, researchers were unable to explore the particularities of each case in depth, which is considered to be a limitation of the present study. In addition, employing a conceptual framework such as sexual trauma may present a bias when interpreting results, thereby limiting understanding of different traumatic experiences or developmental challenges made visible through sandplay. Here, further study is needed. Moreover, the utilization of cameras in the therapeutic space was met with mixed reactions. It would valuable to further analyze the use of recordings as both a research tool and a therapeutic technique. Future research may draw on mixed methodologies (including verbal reporting) to complement results obtained by sandplay, and also evaluate other ludic techniques. It is also possible to explore this phenomenon at different developmental stages with children who have experienced other types of abuse and emotional distress in order to identify similarities and differences that provide greater insight into the phenomenon of creative play.

With regards to the practical implications of study results in clinical practice, sand tray therapy emerges as a useful tool for the treatment of child victims of sexual assault. In this study, it promotes an in-depth analysis of cases over an extended period of time, thereby providing clinicians with a tool that could give insight into how children resignify their experiences and transform and overcome sexual abuse. Finally, we may hypothesize that the creative potential of sand tray therapy is reached once a therapeutic bond is established – a hypothesis that should be addressed in future studies.

## Author Contributions

The work was developed by MT and supervised by CC. CC also contributed to the process of analysis and triangulation of information and the article writing.

## Conflict of Interest Statement

The authors declare that the research was conducted in the absence of any commercial or financial relationships that could be construed as a potential conflict of interest.

## Footnotes

1 In the excepts presented here, “N” is used to identify the child or participant’s response, while “T” is used for the therapist’s response. Hyphens signal observations or clarifications to the text. Finally, at the end of the excerpt, a series of letters is used to identify the participant, which corresponds to the participants identification presented in **Table 2**. Videos are marked “V” and are followed by a number (1, 2, and 3) depending on the phase of treatment.
